# The Role of DmCatD, a Cathepsin D-Like Peptidase, and Acid Phosphatase in the Process of Follicular Atresia in *Dipetalogaster maxima* (Hemiptera: Reduviidae), a Vector of Chagas' Disease

**DOI:** 10.1371/journal.pone.0130144

**Published:** 2015-06-19

**Authors:** Jimena Leyria, Leonardo L. Fruttero, Magalí Nazar, Lilián E. Canavoso

**Affiliations:** Departamento de Bioquímica Clínica, Centro de Investigaciones en Bioquímica Clínica e Inmunología (CIBICI-CONICET), Facultad de Ciencias Químicas, Universidad Nacional de Córdoba, Córdoba, CP 5000, Argentina; UMR INRA/INSA, BF2I, FRANCE

## Abstract

In this work, we have investigated the involvement of DmCatD, a cathepsin D-like peptidase, and acid phosphatase in the process of follicular atresia of *Dipetalogaster maxima*, a hematophagous insect vector of Chagas’ disease. For the studies, fat bodies, ovaries and hemolymph were sampled from anautogenous females at representative days of the reproductive cycle: pre-vitellogenesis, vitellogenesis as well as early and late atresia. Real time PCR (qPCR) and western blot assays showed that DmCatD was expressed in fat bodies and ovaries at all reproductive stages, being the expression of its active form significantly higher at the atretic stages. In hemolymph samples, only the immunoreactive band compatible with pro-DmCatD was observed by western blot. Acid phosphatase activity in ovarian tissues significantly increased during follicular atresia in comparison to pre-vitellogenesis and vitellogenesis. A further enzyme characterization with inhibitors showed that the high levels of acid phosphatase activity in atretic ovaries corresponded mainly to a tyrosine phosphatase. Immunofluorescence assays demonstrated that DmCatD and tyrosine phosphatase were associated with yolk bodies in vitellogenic follicles, while in atretic stages they displayed a different cellular distribution. DmCatD and tyrosine phosphatase partially co-localized with vitellin. Moreover, their interaction was supported by FRET analysis. *In vitro* assays using homogenates of atretic ovaries as the enzyme source and enzyme inhibitors demonstrated that DmCatD, together with a tyrosine phosphatase, were necessary to promote the degradation of vitellin. Taken together, the results strongly suggested that both acid hydrolases play a central role in early vitellin proteolysis during the process of follicular atresia.

## Introduction

Vitellogenesis is a major event in the reproduction of oviparous organisms. In insects, this process involves the fast growth of the oocytes at the expense of the extra-ovarian synthesis of large amounts of yolk protein precursors (YPPs) [1]. In most insect species, the fat body is the main tissue involved in the synthesis of YPPs, being vitellogenin the most quantitatively and physiologically important [2].

Vitellogenin is a phospholipoglycoprotein of high molecular weight that is taken up by the oocytes by receptor-mediated endocytosis and stored as vitellin along with other YPPs in specialized structures termed yolk bodies [3]. During embryogenesis the gradual proteolysis of vitellin, mediated by acid hydrolases, allows to support the energetic demand of the embryonic development [4]. Most of acid hydrolases reported at present are YPPs synthesized in the fat body, which in turn are released to the hemolymph as pro-enzymes and stored in the ovarian follicles associated with yolk bodies [5]. Interestingly, acid hydrolases involved in yolk degradation can also be synthesized in the ovary [4, 6].

Several acid hydrolases such as cathepsin-like peptidases and acid phosphatases involved in the degradation of vitellin during embryogenesis have been characterized in the oocytes of different insect species [6–9]. It has been proposed that during the embryonic development of the triatomine *Rhodnius prolixus*, an acid phosphatase, inorganic polyphosphate and cathepsin D are important regulators of yolk protein degradation [10, 11].

Cathepsin D is a soluble lysosomal aspartic endopeptidase synthesized as pre-pro-cathepsin D. After removal of its signal peptide, pro-cathepsin D is targeted to intracellular vesicular structures. Upon entering acidic compartments, proteolytic processing yields the mature active lysosomal peptidase, which consists of heavy and light chains linked by non-covalent interactions [12]. Cathepsin D is strongly inhibited by pepstatin A and is involved in the degradation and/or activation of proteins, hormones and enzymes, among others [12, 13]. On the other hand, acid phosphatase belongs to a group of enzymes that hydrolyze phosphomonoesters of a wide variety of substrates in an acidic medium. Acid phosphatases are classified into families according to their substrate specificities for phosphorylated serine, threonine or tyrosine residues and all of them are involved in biological functions that are essential for cell homeostasis [14].

In insects, unfavorable physiological or environmental conditions promote changes in the ovarian tissue that led to the degeneration of some follicles to an atretic stage. During this process, known as follicular atresia, some oocytes are resorbed instead of continuing their development to the final formation of the egg. One of the main factors that promote follicular atresia and oocyte resorption (oosorption) is the scarcity or absence of food [15, 16]. In some insects, oosorption is characterized by the release to the hemolymph of intact vitellin or as small peptides and amino acids [17, 18]. Experimental evidence from the mosquito *Culex pipiens pallens* demonstrated that cathepsin B and L-like peptidases, which participate in yolk protein degradation during embryogenesis, are activated early in response to blood deprivation in order to promote follicular atresia and resorption of oocytes [19].

Triatomines or “kissing bugs” are hematophagous insects with relevance in the public health of South and Central America since they are vectors of the parasite *Trypanosoma cruzi*, the etiological agent of Chagas’ disease. Currently, the disease affects about 8 million people in several countries [20]. In triatomine females, each gonotrophic cycle is tightly coupled to the intake of blood meal [21]. Although autogeny has been reported in some species of Triatominae [22], most females need a blood meal to trigger vitellogenesis, which lasts until the end of the oviposition period. However, during starvation or when the amount of ingested blood failed to promote oocyte growth, some ovarian follicles degenerate and become atretic. Although follicular atresia has relevance in the biology of reproduction of Chagas’ disease vectors and may cease if female ingests blood, it has received little attention in insect reproduction studies. The process was described in a cytological study of the ovary of *R*. *prolixus* [23]. Later on, Medeiros et al. [24] reported that atresia of ovarian follicles challenged with non-entomopathogenic fungus led to an increase of peptidase activities, which in turn were involved in degradation of yolk protein content.

*Dipetalogaster maxima*, the largest triatomine species, inhabits the sylvatic environments of Baja California Sur, Mexico [25]. This is an aggressive species that usually takes its blood meal from lizards. However, it has been reported its adaptation to suburban areas, feeding on human or domestic animals [26]. Despite their relatively lower epidemiologic relavence compared to other triatomine vectors, the females of *D*. *maxima*, do to their morphologic characteristics, are excellent models for studying reproductive physiology in triatomines. Under standardized conditions, the anautogenous female takes a large blood meal to elicit vitellogenesis. After the first oviposition period, ovaries enter into a post-vitellogenic stage during which, some terminal follicles become atretic and the oocytes are resorbed [27]. In *D*. *maxima*, morphological changes of ovarian tissue during post-vitellogenesis progress gradually enabling to characterize the events occurring in early and late stages of follicular degeneration [27, 28]. On the contrary, in most insect species, the onset of atresia and oocyte resorption occur very fast, being evidenced only in advanced stages, when most of the oocytes are resorbed [15, 19]. Partial vitellin proteolysis, activation of cathepsin D-like peptidase as well as follicle removal by apoptosis and autophagy are among the main events involved in follicular atresia of *D*. *maxima* [27, 28]. The changes in ovarian nutritional resources found at the atretic stages were also reported in this species [29].

In the present work, the experimental approaches were directed to address the role of DmCatD, a cathepsin D-like peptidase, and acid phosphatase in the process of follicular atresia in the vectors of Chagas’ disease, focusing in their involvement in yolk protein degradation. Employing *D*. *maxima* as a model, we evaluated the expression of DmCatD in the fat body and ovarian tissues at representative days of the reproductive cycle. Enzymatic assays provided evidence about the involvement of both, DmCatD and acid phosphatase in follicular atresia. The class of acid phosphatase present in ovaries was established using specific phosphopeptides as enzymatic substrates and acid phosphatase inhibitors. Immunofluorescence assays revealed the localization of the two acid hydrolases in ovarian follicles. *In vitro* proteolysis assays addressed the role of DmCatD and a tyrosine phosphatase in the degradation of vitellin. Our results strongly suggest that early activation of DmCatD and tyrosine phosphatase is a relevant physiological mechanism that regulates yolk protein degradation during follicular atresia to either, increase female lifespan or sustain younger oocytes until improvement of nutritional conditions.

## Materials and Methods

### Chemicals

Rabbit polyclonal antibody anti-human cathepsin D (sc-10725), rabbit polyclonal antibody anti-human PTP1B (protein tyrosine phosphatase, sc-14021) and MCF7 whole cell lysate were from Santa Cruz Biotechnology (Palo Alto, CA, USA). All commercial antibodies were chosen on the base of their conserved sequences, using for the comparisons the database of insects (taxid:6960) of the National Center for Biotechnology Information (NCBI). Alexa Fluor 488- and 568-conjugated goat anti-rabbit IgG antibodies (Molecular Probes, Carlsbad, CA, USA); Tissue-Tek embedding medium (OCT) (Miles, Elkhart, IN, USA); Fluorsave (Calbiochem, Darmstadt, Germany); Western Lightning Plus-ECL, enhanced chemiluminescence substrate (PerkinElmer, Waltham, MA, USA) and electrophoresis protein standards (New England Biolabs, Ipswich, MA, USA) were from indicated commercial sources. Assay kits for serine/threonine phosphatase (V2460) and tyrosine phosphatase (V2471), as well as MMLV reverse transcriptase were obtained from Promega (Heidelberg, Germany). Primers were from Sigma Genosys (Houston, TX, USA); MasterPure RNA Purification Kit was obtained from Epicenter Biotechnologies (Madison, WI, USA) and Brilliant SYBR Green qPCR Master Mix was a product of Stratagene from Agilent Technologies, Inc. (Santa Clara, California, USA). All other chemicals were from Sigma-Aldrich (St. Louis, MO, USA). The fluorogenic peptide substrate Abz-AIAFFSRQ-EDDnp (Abz, orthoaminobenzoic acid, EDDnp, ethylenediamine-2,4-dinitrophenyl) was a kind gift from Dr. Maria Aparecida Juliano (Universidade Federal de São Paulo, Brazil).

### Insects

Experiments were carried out using insects taken from a colony of anautogenous *D*. *maxima*, maintained at 28°C, 70% relative humidity and 8:16 h light:dark photoperiod. Insects free of *T*. *cruzi* or *Blastochritidia triatomae* were fed on hen blood [30] according to the recommendations of the National Institute of Parasitology (National Health Ministry, Argentina) [31].

Standardized conditions of insect rearing for the study were previously described [27, 28]. Briefly, fifth-instar females were separated from males before feeding. Upon molting, each female was placed in individual containers with two fed males during 48 h. The presence of the spermatophore in the container was considered as evidence of successful mating. Each mated female was maintained in an individual jar until it was able to ingest a blood meal (days 10–12 post-ecdysis), which resulted in a 3.0–5.5 fold increase in the body weight of the insect. Thereafter, egg laying was daily monitored and both, the beginning and the end of the oviposition period, which was confirmed if females did not lay eggs during five consecutive days, were recorded. Females were monitored at least for 32 days after the end of the oviposition (post-vitellogenesis). During post-vitellogenesis females did not receive any further blood meal [27].

Experimental approaches were performed by sampling fat bodies, ovaries and hemolymph at representative days of the reproductive cycle as previously described: (a) pre-vitellogenesis (day 2 post-ecdysis, unfed period); (b) vitellogenesis (days 4–6 post-blood feeding); (c) post-vitellogenesis (days 10–12 and days 30–32 after the end of oviposition for early and late atresia, respectively) [27, 28].

### Ethics Statement

Housing conditions and manipulation of hens followed current protocols of the Centro de Investigaciones en Bioquímica Clínica e Inmunología (CIBICI-CONICET-Universidad Nacional de Córdoba) animal facility, in accordance with the guidelines published by the Canadian Council on Animal Care with the assurance number A5802-01 delivered by the Office of Laboratory Animal Welfare (National Institutes of Health). The protocol was authorized by the Animal Care Committee of CIBICI-CONICET-Universidad Nacional de Córdoba (Exp. 15-UNC-05-54976). According to institutional policy, no specific number of approval was assigned since no infective species, human blood or animal sacrifice were involved in the study. The animal facility at the Centro de Investigaciones en Bioquímica Clínica e Inmunología (CIBICI-CONICET-Universidad Nacional de Córdoba) is a dependency of the Argentine National Ministry of Science (Sistema Nacional de Bioterios, MINCyT, http://www.bioterios.mincyt.gob.ar). Briefly, hens were housed individually in a room at 25°C, with food and water available *ad libitum*, under a light-dark cycle of 12 h each. Cages were cleaned daily and hens’ health was regularly checked by a veterinary physician. Special care was taken in their handling in order to minimize stressful conditions. During the process of bug feeding (30 min, 4 insects/animal), hens were maintained in a special dark room and secured to the table by soft bands. Each of the hens participated in feeding purposes only once in a month.

### Tissue and hemolymph sampling

Ovaries and fat bodies from females at different days of the reproductive cycle were carefully dissected out under cold phosphate buffered saline (PBS, 6.6 mM Na_2_HPO_4_/KH_2_PO_4_, 150 mM NaCl, pH 7.4), using a standard stereoscope with an optic fiber light source.

The hemolymph was individually collected from immobilized females with a Hamilton syringe and placed in ice-cold microtubes in the presence of 10 mM Na_2_EDTA and 5 mM dithiotreitol (DTT) as previously reported [27]. For western blot assays, a mixture of protease inhibitors containing 1 μM aprotinin, 0.5 μM N-α-tosyl-L-lysine chloromethyl ketone (TLCK) and 1 mM benzamidine was added to each sample. All the hemolymph samples were centrifuged at 10,000 *x* g for 5 min at 4°C to remove hemocytes. Thereafter, samples were employed for the biochemical assays, previous protein determination [32].

### RNA extraction and Reverse Transcription/quantitative PCR (RT-qPCR)

Taking into account the role of fat body and ovarian follicular cells in the synthesis of yolk protein precursors, transcriptional levels of DmCatD were determined in fat bodies and ovaries sampled at different stages of the reproductive cycle of *D*. *maxima*. For RNA extraction, dissected tissues of three females were pooled and the MasterPure RNA Purification Kit was used according to the manufacturer´s protocol. To eliminate genomic DNA, samples were treated with DNAse provided in the kit. RNA integrity was evaluated by electrophoresis in a 1% agarose gel. cDNA was synthesized from 2 μg of total RNA by reverse transcription reaction using the MMLV reverse transcriptase protocol. Real time PCR (qPCR) analysis was performed using an ABI Prism 7500 sequence detection system (Applied Biosystems, Foster City, CA, USA) and SYBR green chemistry as described [33]. The 2^−ΔΔCt^ method was used to quantify relative changes in gene expression using 18S ribosomal RNA (18S rRNA) as internal control. Reactions were carried out in triplicate using, for each 15 μl reaction, 5 μl of cDNA template (equivalent to 300 ng of total RNA). For each pair of primers, a dissociation plot resulted in a single peak, indicating that a single cDNA product was amplified (S1A and S1B Fig). Moreover, re\actions were subjected to agarose gel electrophoresis to check amplification specificity. All reactions showed an amplification efficiency higher than 98%. Specific target amplification was confirmed by automatic sequencing (Macrogen, Seoul, Korea). The primers sequences for DmCatD (96 pb) were: sense, 5`-GGTGAAGCACTGCTAGTGTCG-3´ and antisense, 5´-AGCTTGATCTTCGGCAATTG-3´. The primer sequences for the internal control 18S rRNA (104 bp) were: sense, 5´-TCGGCCAACAAAAGTACACA-3´ and antisense, 5´-TGTCGGTGTAACTGGCATGT-3´. The design of primers was based on the available mRNA sequences of related species.

### DmCatD expression by western blot

Fat bodies and ovaries from females dissected at different stages of the reproductive cycle were individually homogenized in buffer Tris-HCl-Na_2_EDTA (50 mM Tris, 1 mM EDTA, 0.1% Triton X-100, pH 7.5) containing a mixture of protease inhibitors (1 μM aprotinin, 0.5 μM TLCK and 1 mM benzamidine). The first centrifugation was performed at 2,500 x *g* for 10 min at 4°C and the floating fat cake and pellet were discarded. The resulting material was subjected to a second centrifugation at 20,000 x *g* for 20 min at 4°C and the supernatants collected and used for western blotting. Protein concentration in tissue homogenates was determined according to Bradford [32].

For western blots, protein extracts from fat body and ovary homogenates as well as hemolymph (40 μg each one) were subjected to 10% SDS-Tris-Tricine gel electrophoresis [34] and then transferred onto a nitrocellulose membrane for 1 h as described elsewhere [35]. The membrane was incubated with Ponceau S and the images were registered. After rinsing with 10 mM Tris-HCl buffer containing 150 mM NaCl (TBS, pH 7.5), the nitrocellulose was blocked with TBS-0.1% Tween 20 containing 5% non-fat milk at room temperature. The antibodies were diluted in TBS-0.1% Tween 20 containing 5% BSA and used in the incubation steps as follows: polyclonal anti-cathepsin D antibody (primary antibody, dilution 1:300, at 4°C, overnight) and HRP conjugated goat anti-rabbit IgG (secondary antibody, dilution 1:2000, for 1 h at room temperature). All incubations were carried out with gentle rocking. The immunoreactive bands were detected by ECL, according to the manufacturer’s instruction. Densitometric analyses of fat bodies and ovaries western blot profiles were performed using ImageJ software. For each lane, the intensity of the bands compatible with pro-DmCatD and DmCatD and the amount of total proteins loaded and stained with Ponceau S were considered (S2A and S2B Fig). MCF7 whole cell lysate derived from the MCF cell line was used as a positive control of western blot assays.

### Measurement of DmCatD activity

The activity of DmCatD peptidase upon the specific synthetic fluorogenic substrate Abz-AIAFFSRQ-EDDnp was assayed either in aliquots of hemolymph or in tissue homogenates (fat bodies or ovaries) containing 30 μg of proteins according to the protocol previously reported [27]. Reactions were monitored every 11 s for 30 min in an F-Max fluorometer (Molecular Devices Inc., Silicon Valley, CA, USA) with 320/430 nm excitation/emission filters. Results were expressed as relative units of fluorescence (RUF)/μg protein/min.

### Measurement of acid phosphatase activity

The activity of acid phosphatase in ovarian tissues was assayed measuring the hydrolysis of *p*-nitrophenyl phosphate (*p*NPP) to *p*-nitrophenol (*p*NP) as reported previously [28]. Briefly, the tissues were individually homogenized in 20 mM sodium acetate buffer pH 4.0 containing 10 mM DTT, 10 mM Na_2_EDTA, 2 mM phenylmethylsulfonyl fluoride (PMSF), 0.01 mM pepstatin and 50 μM soybean trypsin inhibitor (SBTI). Homogenates were then centrifuged at 14,000 x *g* for 5 min at 4°C and the resulting supernatants were subjected to protein quantification [32]. Enzymatic assays were performed using 5 mM *p*NPP as substrate and 30 μg of protein homogenates as enzyme source. The amount of *p*NP released into the medium was registered at 405 nm. Specific activity was expressed as nmol *p*NP/mg protein/min. Acid phosphatase activity was also determined by pre-incubating the homogenates from females at early atresia with NaF (an inhibitor of serine/threonine phosphatases), Na_3_VO_4_ (an inhibitor of tyrosine phosphatases) or with Na^+^/K^+^ tartrate (a broad spectrum phosphatase inhibitor) at a final concentration of 1 mM each (30 min, at 37°C). Thereafter, assays were carried out as previously stated.

On the other hand, enzymatic assays using specific synthetic phosphopeptide substrates were performed to determine if the activity of acid phosphatase found in ovarian tissue during follicular atresia corresponded to either, a protein tyrosine phosphatase or a protein serine/threonine phosphatase. The assays were carried out on microplates provided in the commercial kits for serine/threonine phosphatase and tyrosine phosphatase following their manufacturer’s instructions. Three-five μg of protein extracts from ovaries at early and late atresia were added to the incubation media. The generation of free phosphate was determined by measuring the absorbance of a complex formed by phosphate, molybdate, and malachite green using the Multi-Mode Microplate Reader Sinergy HT (BioTek Instruments, Winooski, VT, USA) at 600 nm [36]. The specific activity was expressed as pmol phosphate/μg protein/min. To further characterize the class of acid phosphatase, aliquots of ovarian homogenates at both atretic stages were pre-incubated for 30 min with NaF, Na_3_VO_4_ or Na^+^/K^+^ (1 mM each, 30 min at 37°C). Then, the enzymatic assays for tyrosine or serine/threonine phosphatases were performed as described above.

### Localization of DmCatD and acid phosphatase in ovarian tissue

Immunofluorescence assays for DmCatD and acid phosphatase were directed to assess their localization in ovarian tissue, particularly in oocytes, due to the proposed physiological role of acid hydrolases on vitellin degradation. For the assays, ovaries from females in vitellogenesis and early follicular atresia sampled as described previously were fixed in 4% paraformaldehyde in PBS, transferred to sucrose/PBS and then embedded in OCT and frozen in liquid nitrogen [28]. Thereafter, tissue cryosections (8 μm thick) were obtained with a Cryotome E Cryostat (Thermo Fisher Scientific Inc., Waltham, MA, USA) and placed onto poly-L-lysine-treated glass slides. For the assays, the slides were blocked with 1% BSA, 0.1% Triton X-100, 5% fetal bovine serum in PBS during 1 h, and then incubated overnight at 4°C with the anti-cathepsin D antibody (dilution 1:100) or the anti-tyrosine phosphatase antibody (dilution 1:50). Slides were washed three times with PBS and then incubated for 1 h at 37°C with either Alexa Fluor 568-conjugated goat anti-rabbit IgG antibody (for DmCatD, dilution 1:1000) or Alexa Fluor 488-conjugated goat anti-rabbit IgG antibody (for tyrosine phosphatase, dilution 1:1000). In all cases, the antibodies were diluted in PBS containing 1% BSA. Finally, the slides were rinsed with PBS, air-dried, mounted in Fluorsave and viewed with an Olympus Fluoview 300 laser scanning confocal microscope (Olympus, Tokyo, Japan) equipped with a 543 and 488 nm lasers.

### Anti-vitellin antibody and labeling with FITC

The polyclonal anti-vitellin antibody was obtained by inoculating New Zealand rabbits with purified vitellin as described previously [37]. For co-localization assays, anti-vitellin antibody was labeled with fluorescein isothiocyanate (anti-Vt-FITC, 5 mg/ml) in dimethyl sulfoxide according to Hermanson [38].

### Co-localization of DmCatD and acid phosphatase with vitellin in ovarian tissue

These set of studies were directed to obtain evidence about co-localization in yolk bodies of acid hydrolases and vitellin, which in turn would be of functional relevance in the process of yolk protein degradation during atresia. For co-localization assays, ovarian cryosections from vitellogenic females were blocked and then subjected to indirect immunofluorescence assays for DmCatD and tyrosine phosphatase as stated above. After rinsing with PBS, the tissue cryosections were incubated with anti-Vt-FITC in PBS/1% BSA (dilution 1:40) for 1 h at 37°C. Finally, the slides were rinsed with PBS and processed for laser confocal microscopy (Olympus Fluoview 300) as previously described.

In another set of experiments, after the immunofluorescence protocol was performed as described above, the potential interaction of both acid hydrolases with vitellin in the yolk bodies of vitellogenic oocytes was tested by the fluorescence resonance energy transfer (FRET) method [39]. In the fluorescent microscopy, FRET was detected by comparing the donor fluorescence intensity in the same sample before and after destroying the acceptor by photobleaching. If FRET was present, a resultant increase in donor fluorescence will occur on photobleaching of the acceptor. Experiments were performed in an Olympus Fluoview 300 laser scanning confocal microscope, applying a 15 s pulse of high-intensity laser at 543 nm to bleach the fluorescent signal of the enzymes (acceptors). If any interaction leading to energy transfer to a donor (vitellin) were present in the yolk bodies, photobleaching of the acceptor would lead to an increase in the fluorescent signal of the donor molecule because it would no longer be quenched by the acceptor [39]. Results were analyzed by processing a series of pre-bleaching and post-bleaching images, using the FluoView v5.0 software.

### *In vitro* vitellin proteolysis assays

In order to determine the participation of DmCatD and acid phosphatase in the degradation of vitellin during follicular atresia, *in vitro* proteolysis assays were performed with purified vitellin as substrate [37] and, as enzyme source, protein homogenates from ovaries at early follicular atresia obtained as described for western blot assays.

The pH dependence of *in vitro* vitellin proteolysis was analyzed by incubating 30 μg of vitellin and 70 μg of protein homogenates at 37°C during 12 h using the buffer systems containing 200 mM NaCl and 5 mM Na_2_EDTA as follows: 50 mM ammonium formate (for pH 3.0 and 3.5), 50 mM sodium acetate (for pH ranging from 4.0 to 5.0) and 50 mM sodium phosphate (for pH 6.0 and 7.0). Reactions were stopped by the addition of SDS-PAGE sample buffer and then boiled for 5 min [10, 40]. Aliquots containing 50 μg of the reaction mixture were fractionated by SDS-PAGE (7.5%) and then subjected to western blot. The main subunits of vitellin (Mr ~170 kDa and 174 kDa, detected as a single immunoreactive band) and its proteolytic fragments were probed with anti-vitellin antibody (dilution 1:5000) as previously described [27]. Bands were visualized by ECL.

Inhibition of vitellin proteolysis *in vitro* was analyzed by performing the assays according to conditions stated above, in a reaction medium containing 50 mM sodium acetate, 200 mM NaCl, 5 mM Na_2_EDTA (pH 4.0), and in the presence or absence of acid phosphatase inhibitors (2 mM Na_3_VO_4_ or 10 mM Na^+^/K^+^ tartrate) or an specific inhibitor of aspartic peptidases (80 μM pepstatin A). Controls containing aliquots of ovarian homogenate and without addition of purified vitellin were performed to rule out a significant contribution of vitellin by atretic ovaries to the *in vitro* system.

### Statistical analyses

The results are presented as the mean ± Standard Error of the Mean (SEM) from at least three independent experiments, including at each point the ovaries, fat body and hemolymph from females (individuals or pools) as stated in each section. Graphs and statistical tests were performed using GraphPad Prism 6.0 and GraphPad Instat 3.0. (GraphPad Software, San Diego, CA, USA). All datasets passed normality and homoscedasticity tests. Multiple group analysis was conducted by one-way ANOVA. The Student-Newman-Keuls multiple-comparisons test was used as a post-test. A *P* value < 0.05 was considered statistically significant.

## Results

### DmCatD expression in fat body and ovarian tissue

In order to analyze the gene expression of DmCatD in fat bodies and ovarian tissues of *D*. *maxima* females at different stages of the reproductive cycle, its transcription profile by qPCR and protein expression by western blot were assessed.

**Fig 1:**
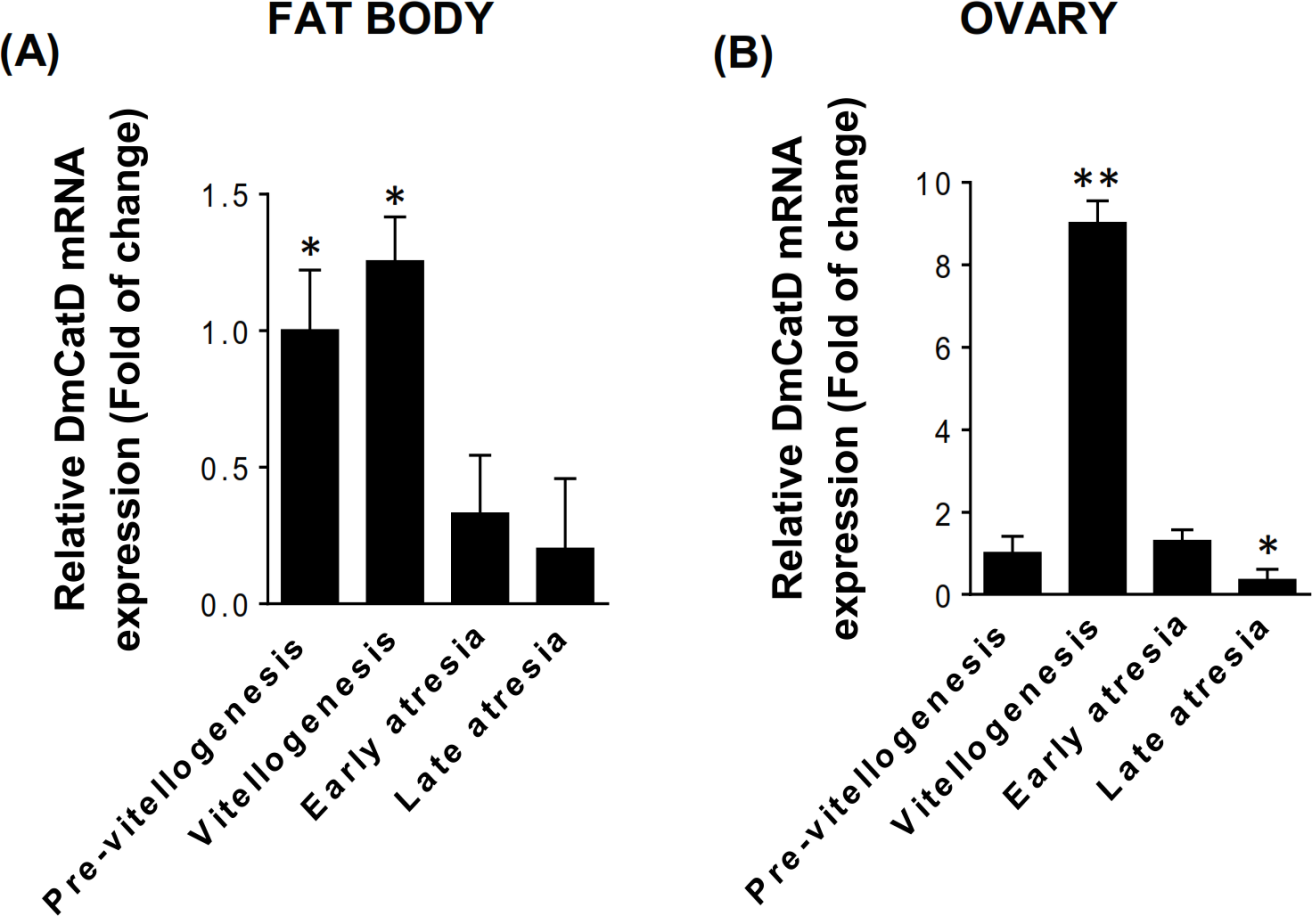
Relative DmCatD mRNA quantification evaluated by RT-qPCR. Total RNA was extracted from fat bodies **(A)** and ovaries **(B)** of females at different stages of the reproductive cycle. Quantification of 18S ribosomal RNA was used as internal control. Results expressed as mean ± SEM indicate the fold of change respect to the mRNA levels at pre-vitellogenesis (n = 3). (A), *P<0.01 vs. early and late atresia. (B), *P<0.05 vs. pre-vitellogenesis and early atresia; **P<0.001 vs. pre-vitellogenesis, early and late atresia.

A single PCR product of the expected size and sequence was amplified (S3A–S3C Fig). When a BLAST analysis was performed (Basic Local Alignment Search Tool) [41] using the default setting, the best match retrieved (89% identity) was the cathepsin D (mRNA) from *Triatoma infestans* (GenBank: GQ871498.1), a related triatomine species. As shown in Fig 1, DmCatD transcripts were detected in both tissues. However, in the fat body, the highest mRNA levels were found at pre-vitellogenesis and vitellogenesis. On the other hand, DmCatD mRNA was upregulated in vitellogenesis when compared to that from pre-vitellogenic and atretic ovaries (Fig 1A). At late atresia, when all ovarioles had terminal follicles in advanced degeneration and oocytes are resorbed, DmCatD mRNA levels were downregulated in ovarian tissue (Fig 1B). Similar amplification patterns were observed in fat bodies and ovarian tissues when conventional PCR assays were performed (S4A and S4B Fig).

**Fig 2:**
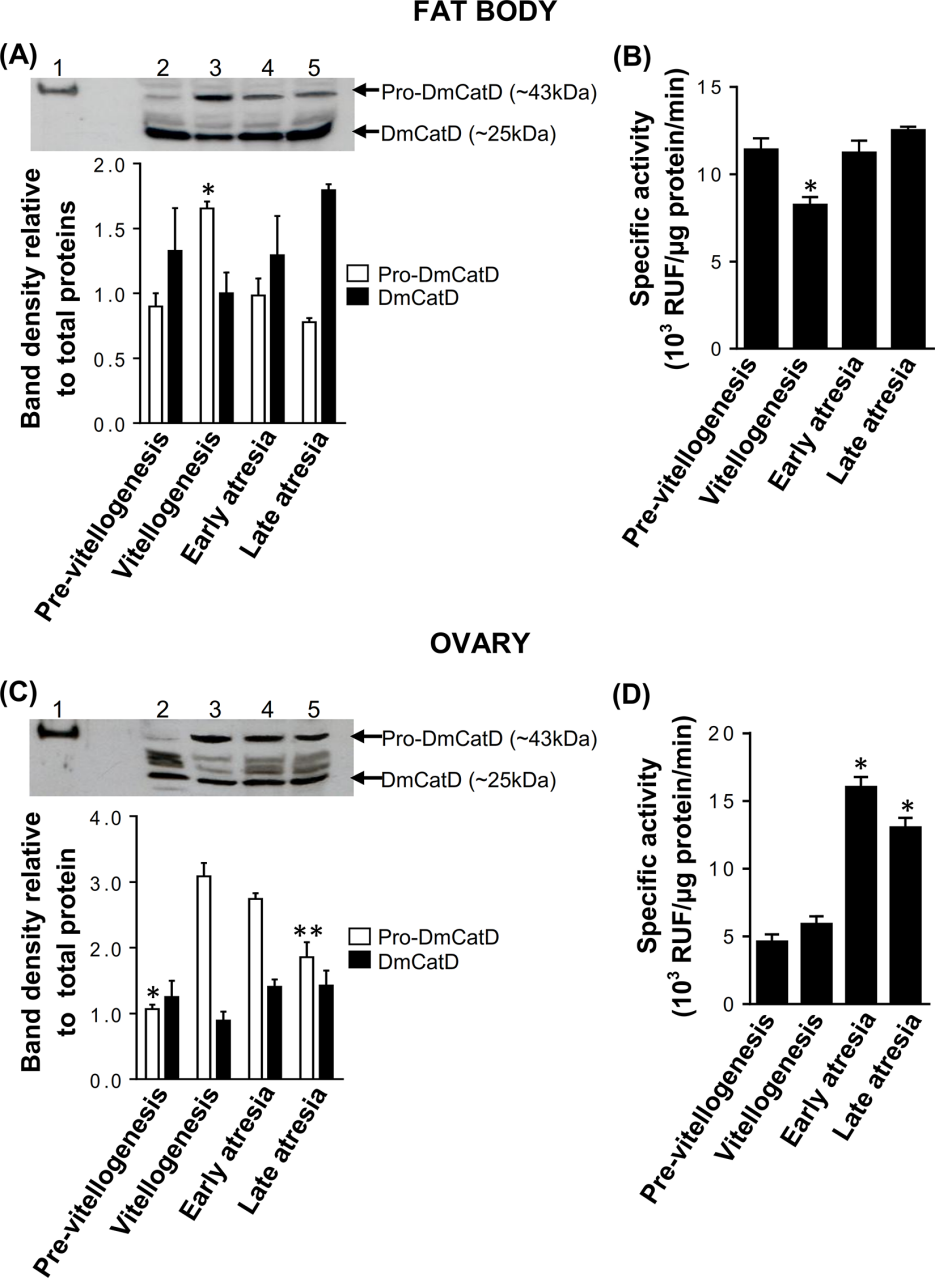
Protein expression and activity of DmCatD in fat bodies and ovaries of *D*. *maxima*. The expression of pro-DmCatD and DmCatD was analyzed by western blot (A and C, top panels). Homogenates of fat body **(A)** and ovarian tissue **(C)** obtained at pre-vitellogenesis, vitellogenesis, early and late follicular atresia (lanes 2–5 respectively, 40 μg each) were probed with a polyclonal anti-cathepsin D antibody. As a positive control, 0.2 μg of whole cell lysate of MCF7 was loaded in the lane 1. The arrows indicate pro-DmCatD (~43 kDa) and DmCatD (~25 kDa). The western blots shown were a representative experiment of three independent assays. Densitometric analyses of three independent western blots (A and C, bottom panels) were performed taking into account the intensity of the bands compatible with pro-DmCatD or DmCatD detected in each lane and the amount of total proteins loaded and stained with Ponceau S. Results are expressed as mean ± SEM (n = 3). (A), *P<0.01 vs. pre-vitellogenesis, early and late atresia; (C), *P<0.001 vs. vitellogenesis, early and late atresia; **P<0.001 vs. vitellogenesis and early atresia. **(B** and **D)**, activity of DmCatD in fat bodies (B) and ovarian homogenates (D) throughout the reproductive cycle. The reactions were performed using 10–30 μg of protein homogenates according to conditions stated in Material and Methods. Results are expressed as relative units of fluorescence (RUF)/μg protein/min and are the mean ± SEM (n = 3). (B), *P<0.05 vs. pre-vitellogenesis, early and late atresia. (D), *P<0.001 vs. pre-vitellogenesis and vitellogenesis.

The changes in the expression of DmCatD protein in fat bodies and ovaries were evaluated by western blot, employing a commercial anti-cathepsin D antibody that recognizes both, the immature proenzyme (pro-DmCatD) and the active form of cathepsin D (DmCatD). Thus, in both tissues, two main immunoreactive bands of molecular weights compatible with pro-DmCatD (~43 kDa) and DmCatD (~25 kDa) were detected at all reproductive stages. Minor faint bands corresponding to either proteolytic fragments or nonspecific immunoreactive signals were also detected (Fig 2A and 2C). Densitometric analyses performed on western blot patterns showed that in fat bodies and ovaries, expression of pro-DmCatD was maximal at vitellogenesis. Pro-DmCatD protein expression then decreased in both tissues at early and late atresia, in coincidence with increased levels of DmCatD (Fig 2A and 2C). These results were in agreement with the increasing levels of DmCatD activity found in fat bodies and ovaries in early and late atresia (Fig 2B and 2D).

### DmCatD in the hemolymph

**Fig 3:**
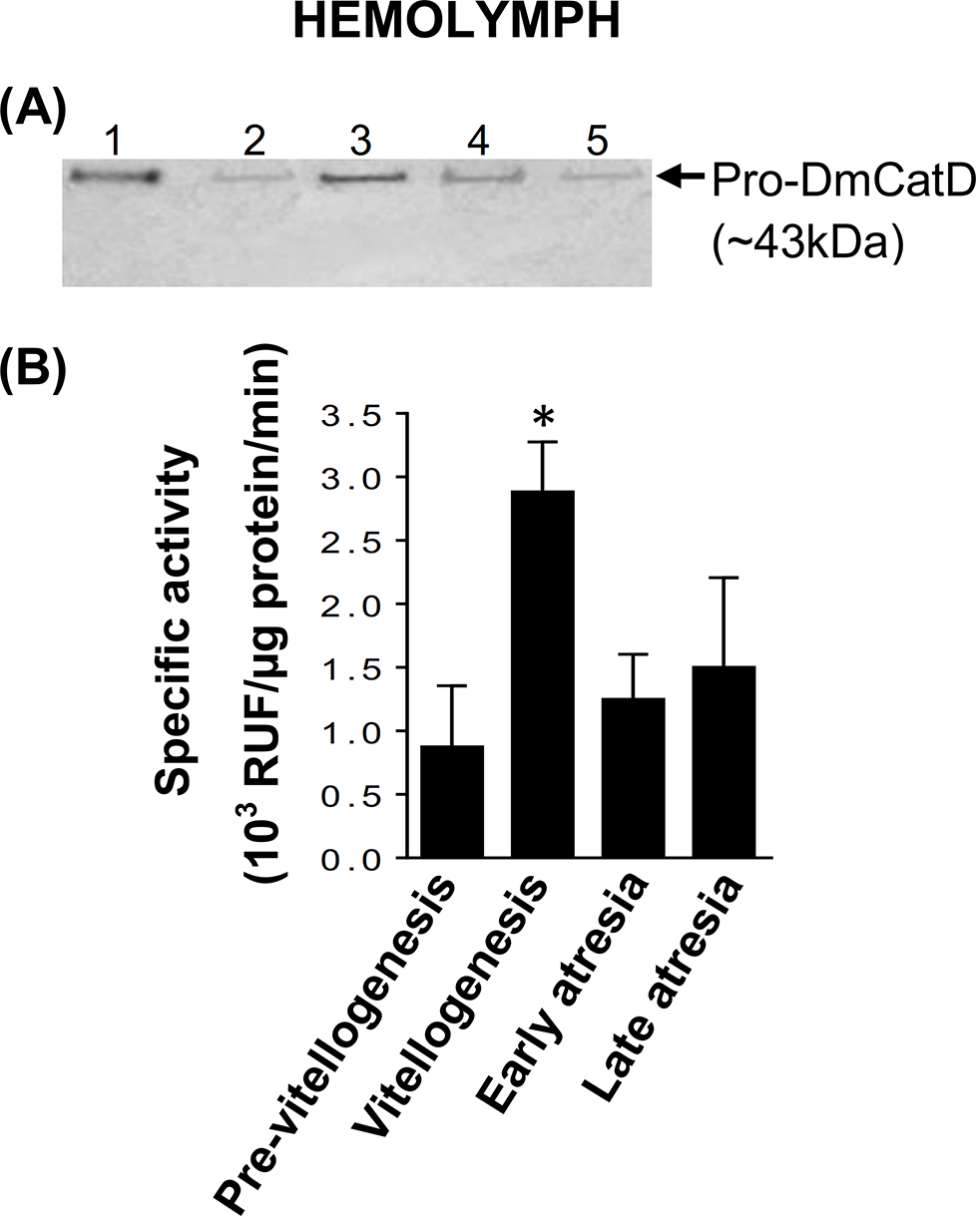
Protein expression and activity levels of DmCatD in the hemolymph of *D*. *maxima*. **(A)**, western blot was performed to evaluate the expression of pro-DmCatD and DmCatD. The hemolymph was obtained at pre-vitellogenesis, vitellogenesis, early and late follicular atresia (lanes 2–5 respectively, 40 μg each). In lane 1, 0.2 μg of whole cell lysate of MCF7 was used as a positive control. The arrow indicates pro-DmCatD (~43 kDa). The western blot shown was a representative experiment of three independent assays. **(B)**, specific activity of DmCatD in the hemolymph. Assays were performed as stated in Materials and Methods. Results expressed as relative units of fluorescence (RUF)/μg protein/min are the mean ± SEM (n = 3). *P<0.05 vs. pre-vitellogenesis, early and late atresia.

When DmCatD was analyzed in the hemolymph of *D*. *maxima* females at different stages of the reproductive cycle by western blot, only the immunoreactive band compatible with pro-DmCatD was detected. Western blot showed low levels of pro-DmCatD at pre-vitellogenesis and increased levels of the protein at vitellogenesis and early atresia (Fig 3A, lanes 2–4). At late atresia, the amount of pro-DmCatD in the hemolymph noticeably decreased, being observed as a faint immunoreactive band (Fig 3A, lane 5).

Although in the hemolymph no immunoreactive band compatible with DmCatD was detected in our experimental conditions, all the hemolymph samples obtained from females at different stages of the reproductive cycle displayed activity upon a specific fluorogenic substrate of DmCatD. The activity was significantly high at vitellogenesis (Fig 3B). However, it is noteworthy that the activity levels of DmCatD in the hemolymph were lower than those registered in fat bodies and ovarian tissues (Fig 2B and 2D).

### Acid phosphatase activity in ovarian tissue

Acid phosphatase activity upon yolk proteins seems to increase their susceptibility to proteolysis during embryogenesis [42]. In addition, follicular atresia in *D*. *maxima* is characterized by partial vitellin proteolysis and significant activity levels of acid phosphatase in ovaries [27, 28]. In this context, we have investigated the class of acid phosphatase present in ovarian tissue homogenates, focusing in the atretic stages.

**Fig 4:**
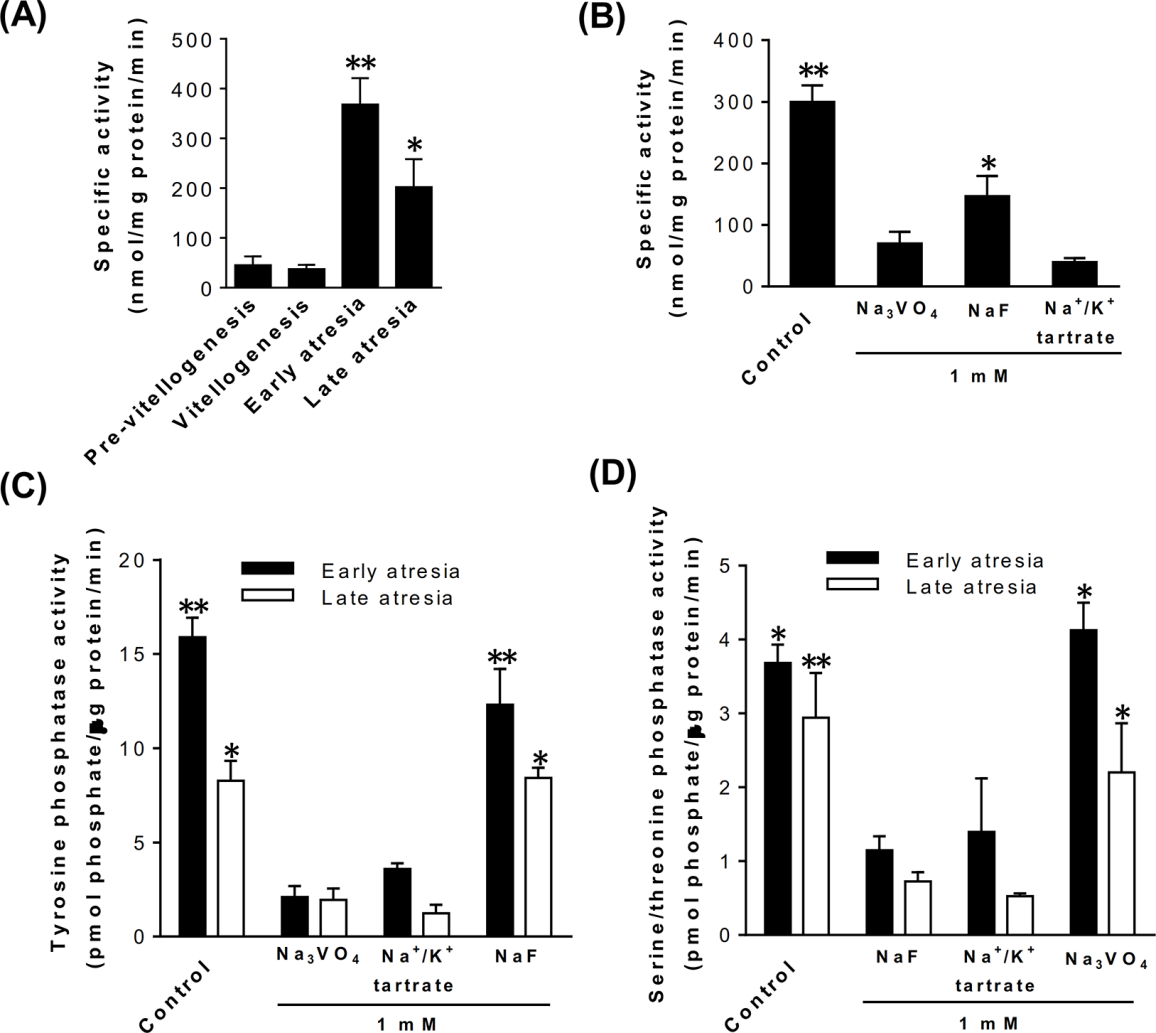
Activity of acid phosphatase in the ovarian tissue of *D*. *maxima*. **(A)**, the enzymatic assays were performed using 30 μg of ovarian homogenates from females at representative days of the reproductive cycle and measuring the amount of *p*-nitrophenol (*p*NP). The results are expressed as specific activity (nmol of *p*NP/mg protein/min) and each point represents the mean ± SEM (n = 3). *P<0.05 vs. pre-vitellogenesis, vitellogenesis and early atresia; **P<0.001 vs. pre-vitellogenesis and vitellogenesis. **(B),** the effect on acid phosphatase activity of several inhibitors was analyzed by pre-incubating protein homogenates of ovaries at early atresia with Na_3_VO_4_ (inhibitor of tyrosine phosphatase), NaF (inhibitor of serine/threonine phosphatase) or Na^+^/K^+^ tartrate (a broad spectrum phosphatase inhibitor). *P<0.05 vs. Na_3_VO_4_ and Na^+^/K^+^ tartrate; **P<0.001 vs. Na_3_VO_4_, NaF and Na^+^/K^+^ tartrate. Specific activity of tyrosine phosphatase **(C)** and serine/threonine phosphatase **(D)** in ovarian homogenates from females at early and late atresia. The assays were performed using specific phosphopeptides as substrates either in the presence or in the absence of NaF, Na_3_VO_4_ or Na^+^/K^+^ tartrate. The specific activities are expressed as pmol phosphate/μg protein/min. Each point represents the mean ± SEM (n = 3). (C), activity in early atresia: **P<0.001 vs. Na_3_VO_4_ and Na^+^/K^+^ tartrate; activity in late atresia: *P<0.05 vs. Na_3_VO_4_ and Na^+^/K^+^ tartrate. (D), activity in early atresia: *P<0.01 vs. NaF and Na^+^/K^+^ tartrate; activity in late atresia: *P<0.01 vs. NaF and Na^+^/K^+^ tartrate; **P<0.001 vs. NaF and Na^+^/K^+^ tartrate.

In the assays using *p*-NPP as substrate, significantly higher activities of acid phosphatase were detected at early and late follicular atresia compared to pre-vitellogenesis and vitellogenesis, although the highest activity was found at days 10–12 post-vitellogenesis (early atresia). Homogenates of ovaries sampled at pre-vitellogenic and vitellogenic stages displayed similar low levels of acid phosphatase activity (Fig 4A). Moreover, in homogenates of tissues at early atresia, the high activity upon *p*-NPP was strongly inhibited by Na_3_VO_4_ (an inhibitor of tyrosine phosphatases, ~77% inhibition) and Na^+^/K^+^ tartrate (a broad spectrum phosphatase inhibitor, ~87% inhibition). Inhibition by NaF, an inhibitor of serine/threonine phosphatases, was also observed although to lesser extent (~51%, Fig 4B). When the inhibitors were tested at higher concentrations, no significant changes on their inhibitory effect were observed. These results strongly suggested that the main responsible of the high acid phosphatase activity found in ovaries at atretic stage was a tyrosine phosphatase.

To further determine the class of acid phosphatase present in atretic ovarian tissues, enzymatic assays employing specific phosphopeptides as substrates of tyrosine phosphatase and serine/threonine phosphatase were performed (Fig 4C and 4D). Results showed that at early atresia, the tyrosine phosphatase activity levels were approximately twice higher than those registered at late atresia. The high activity levels of tyrosine phosphatase in atretic homogenates were significantly inhibited by Na_3_VO_4_ (~87% and 76% inhibition in early and late follicular atresia, respectively) whereas no significant inhibition by NaF (a serine/threonine phosphatase inhibitor) was observed (Fig 4C). However, treatment of homogenates with NaF inhibited the activity of serine/threonine phosphatase (~69% and 78% inhibition in early and late atresia, respectively). No effect of Na_3_VO_4_ on the activity of this class of acid phosphatase was observed (Fig 4D). As expected, tyrosine and serine/threonine phosphatase activities were significantly inhibited with Na^+^/K^+^ tartrate, a broad spectrum phosphatase inhibitor.

### Localization of DmCatD and acid phosphatase in ovarian tissue

**Fig 5:**
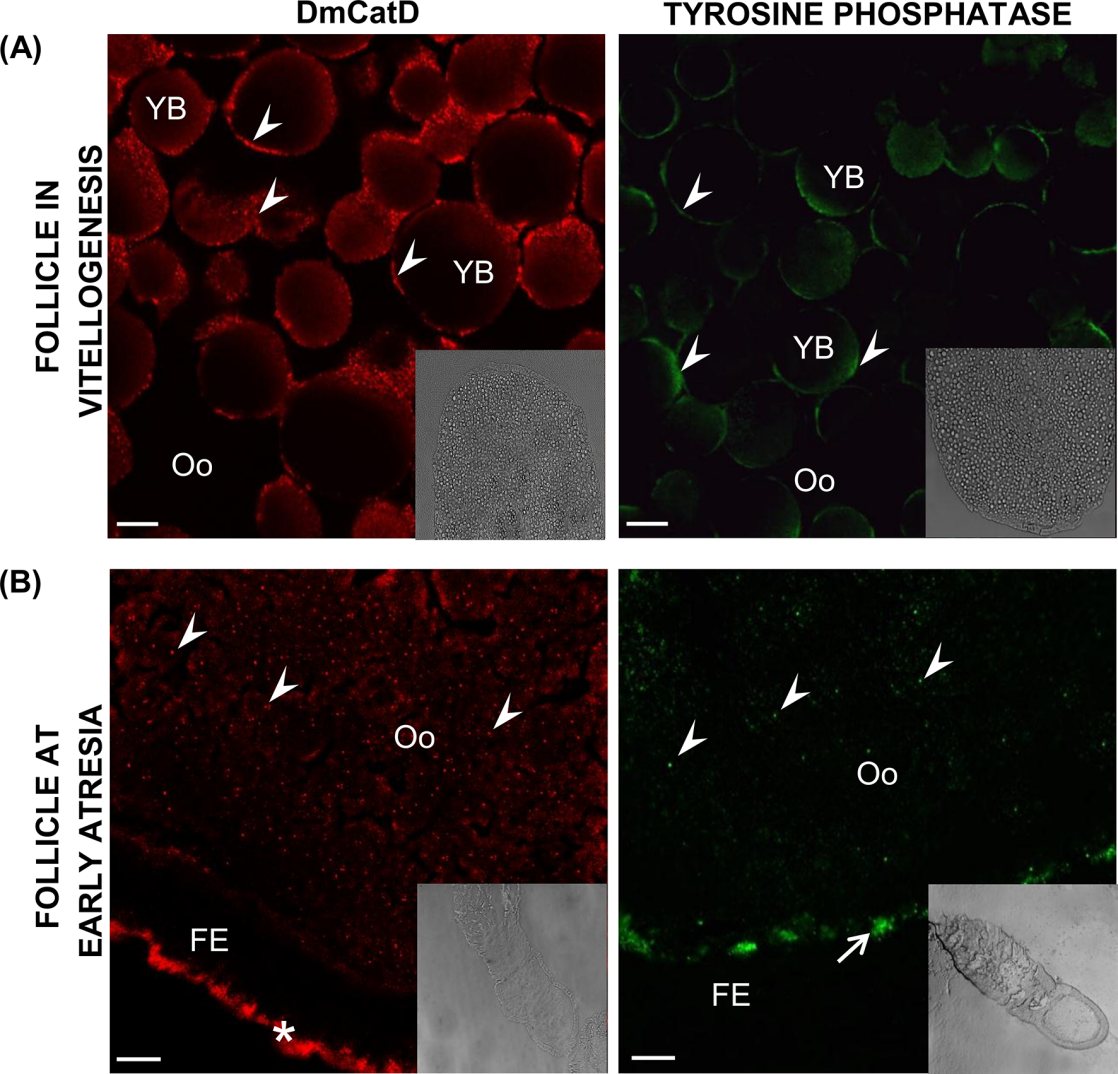
DmCatD and tyrosine phosphatase localization in ovarian follicles by indirect immunofluorescence. Ovaries from females at vitellogenesis and early atresia were incubated with an anti-cathepsin D antibody (red) or with an anti-tyrosine phosphatase antibody (green) and processed as stated in Materials and Methods. Cryostat sections were analyzed by scanning laser confocal microscopy. **(A),** in vitellogenic follicles, the fluorescent signal for both acid hydrolases was associated to yolk bodies (arrowheads). **(B),** at early atresia, oocytes of follicles undergoing incipient degeneration displayed a homogeneous punctate fluorescent pattern for DmCatD and tyrosine phosphatase (arrowheads). At early atresia, DmCatD was also observed in the basal area of follicular epithelium (asterisk), whereas tyrosine phosphatase signal was localized in the perioocytic space of terminal follicles (arrow). Insets correspond to DIC images at lower magnifications. Oo, oocyte; FE, follicular epithelium; YB, yolk bodies. Bars: 10 μm. Similar results were observed after examination of 4–5 ovarioles per ovary in each separate experiment (n = 3).

Localization of DmCatD and tyrosine phosphatase in ovarian tissues at different stages of the reproductive cycle of *D*. *maxima* was assessed by indirect immunofluorescence assays, using commercial antibodies. In vitellogenic follicles, the fluorescent signal for both acid hydrolases was clearly associated to yolk bodies (Fig 5A). By contrast, in early atresia, oocytes of follicles undergoing incipient degeneration displayed a homogeneous punctate fluorescent pattern for DmCatD and tyrosine phosphatase (Fig 5B). Moreover, in early atresia, DmCatD was also observed in the basal area of the follicular epithelium, whereas tyrosine phosphatase signal was localized in the perioocytic space of terminal follicles (Fig 5B).

Additionally, DmCatD was evaluated in fat bodies sampled from females at vitellogenesis and early follicular atresia. At both stages DmCatD showed a fluorescent punctuated pattern homogeneously distributed thorough the cytoplasm of the trophocytes but the intensity of the signal was less evident at early atresia (S5 Fig).

### Co-localization and interaction of DmCatD and acid phosphatase with vitellin

**Fig 6:**
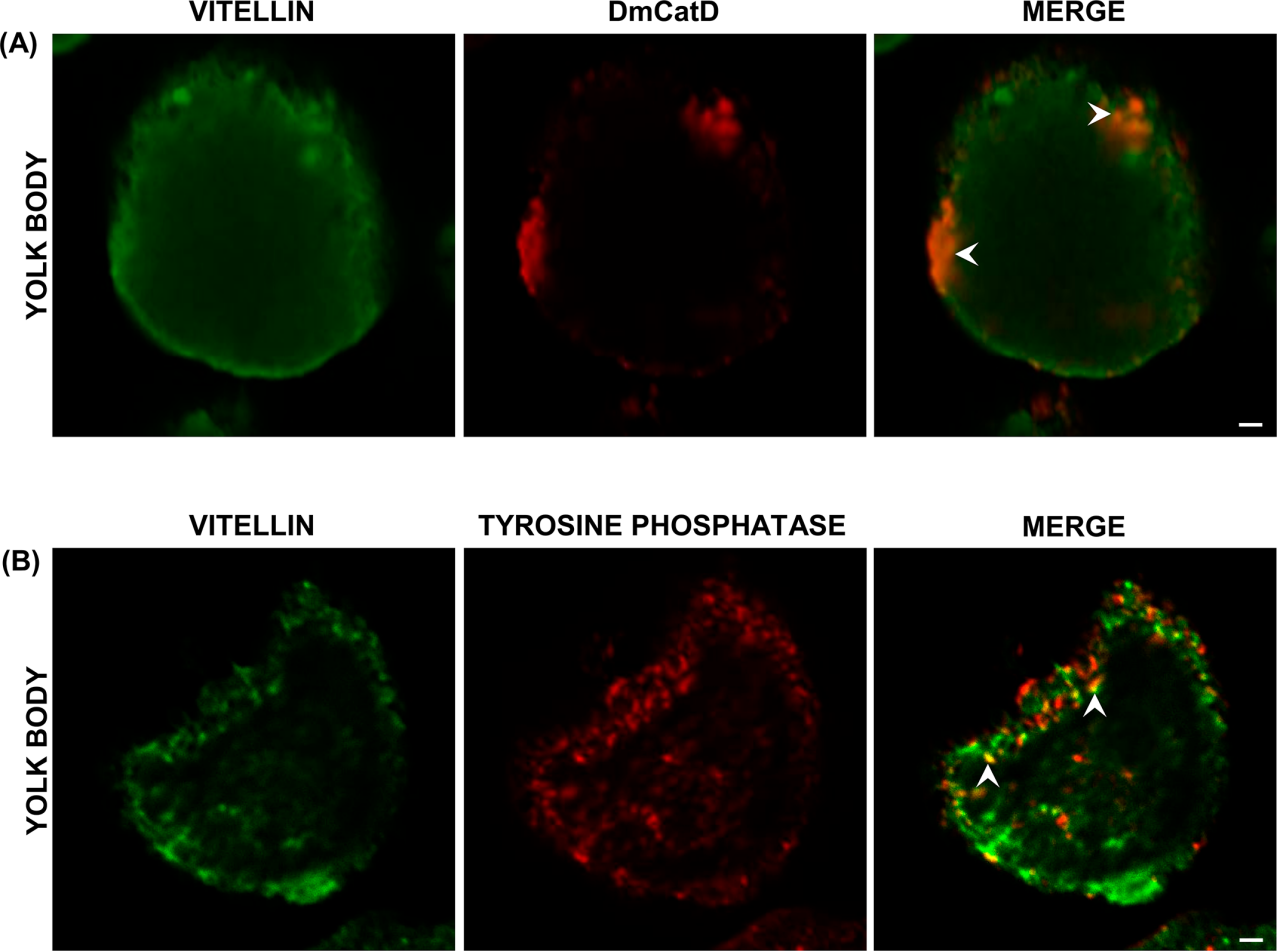
Co-localization of DmCatD and tyrosine phosphatase with vitellin in yolk bodies of vitellogenic oocytes. Vitellogenic ovaries were processed for immunofluorescence as stated in Materials and Methods. **(A-B),** the vitellin (Vt) signal is displayed in green and the enzyme signal (DmCatD or tyrosine phosphatase), in red. In merged images, the partial co-localization of Vt/DmCatD (A) and Vt/tyrosine phosphatase (B) is indicated with arrowheads. Similar results were obtained in three separate experiments. Bars: 2 μm.

The co-localization of DmCatD and tyrosine phosphatase with vitellin was further investigated by immunofluorescence, analyzing yolk bodies of vitellogenic oocytes. Results showed a partial co-localization of both enzymes with vitellin (Fig 6, merge images). However, it seems that such co-localization of DmCatD and vitellin was restricted to some peripheral areas of yolk bodies (Fig 6A, merge) whereas a more heterogeneous pattern of co-localization was observed between tyrosine phosphatase and vitellin (Fig 6B, merge).

**Fig 7:**
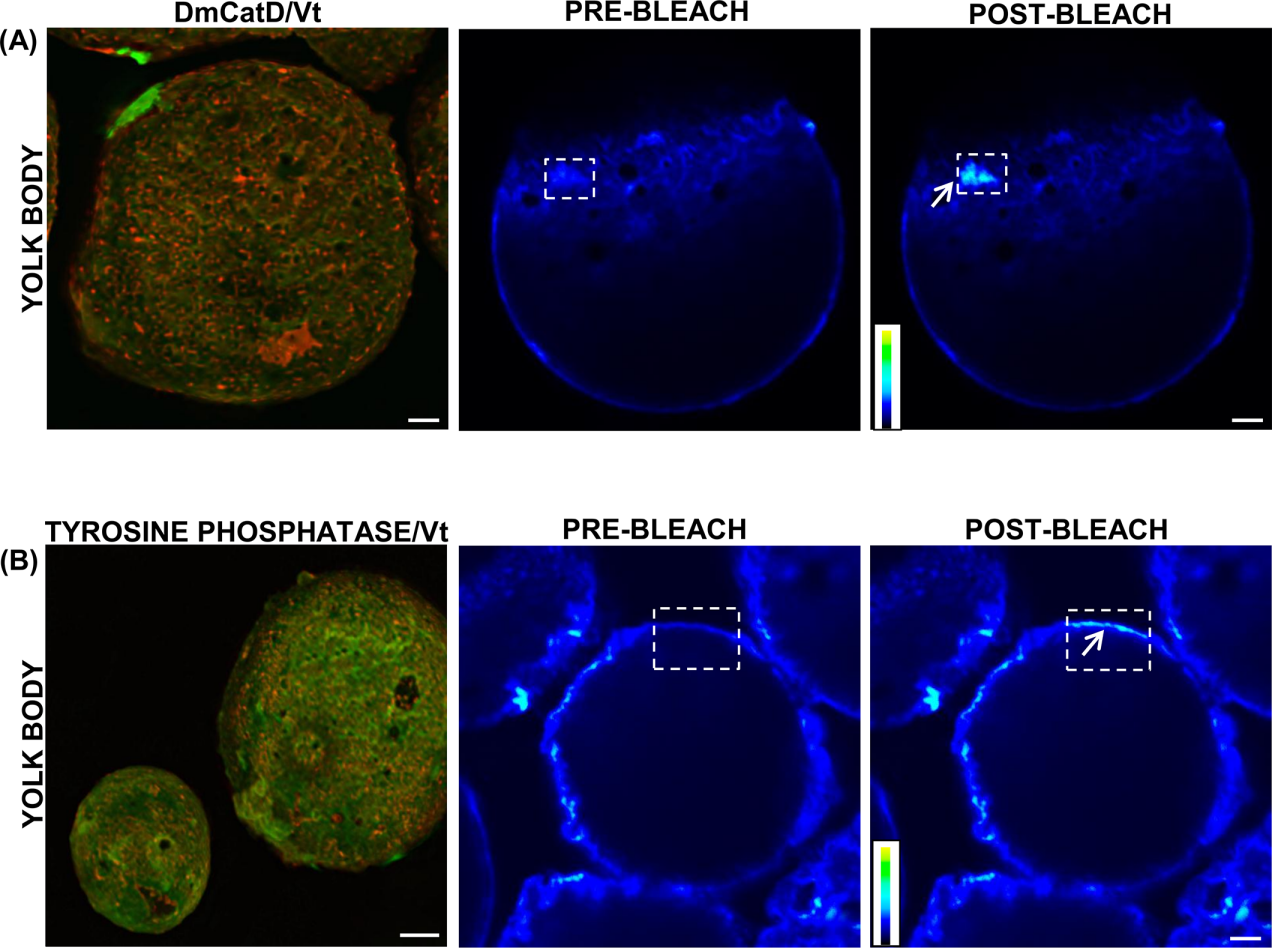
Interaction of DmCatD and tyrosine phosphatase with vitellin in yolk bodies of vitellogenic oocytes. Vitellogenic ovaries were processed for immunofluorescence as stated in Materials and Methods. Images at the left of the panels **(A)** and **(B)** show the Z-projections calculated from maximum intensity signals derived from all focal planes of the image stacks for vitellin (Vt, green) and DmCatD or tyrosine phosphatase (both red). Donor images (Vt signal) pre- and post-photobleaching of the corresponding acceptors, DmCatD and tyrosine phosphatase, show FRET. The white boxes indicate the pre- and post-bleaching areas. Each of the arrows point out the increase in the fluorescence intensity of Vt. A gray scale image pseudocolored with the color gradient is shown within each image to enhance the visualization of the increase in the fluorescence intenstity. Similar results were obtained in 3 separate experiments. Bars: 5 μm for image on the left panel (B) and 2 μm for the other pictures.

On the other hand, when FRET was used to test the interaction between both acid hydrolases and vitellin, a resultant increase in donor green fluorescence (vitellin) was observed after photobleaching of the red fluorescence of the acceptors (DmCatD or tyrosine phosphatase) (Fig 7A and 7B, post-bleach). These results indicated that both enzymes and vitellin were at a distance of 1–10 nm, supporting their potential interaction.

### Involvement of DmCatD and acid phosphatase in the proteolysis of vitellin

In *D*. *maxima*, high activity levels of acid hydrolases were detected in homogenates of atretic ovaries. In addition, the process of follicular atresia in this species was associated to vitellin degradation [27]. Therefore, *in vitro* proteolysis assays using purified vitellin and homogenates of ovaries at early atresia as the enzyme source were conducted to establish the participation of DmCatD and acid phosphatase in vitellin degradation.

**Fig 8:**
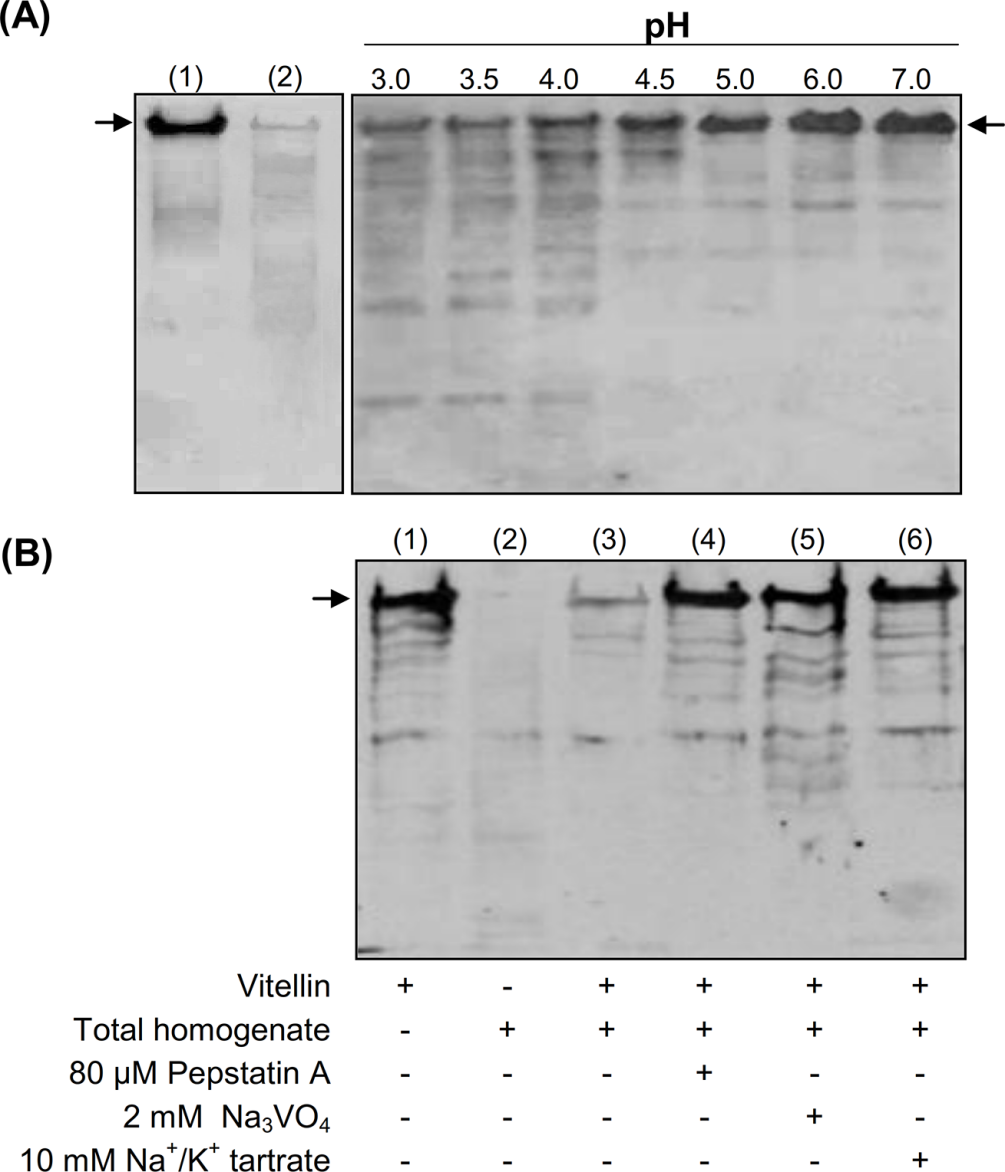
pH dependence and effect of DmCatD and acid phosphatase inhibitors on vitellin (Vt) proteolysis. Homogenates from atretic ovaries were used as the enzyme source and purified Vt as substrate. **(A),** left panel, purified Vt and homogenates of ovarian tissues without incubation (lane 1 and 2, respectively). Right panel, *in vitro* pH dependence of Vt proteolysis. The reaction was carried out for 12 h at 37°C in different reaction buffers, according to the pH evaluated (3.0 to 7.0). In all cases, Vt degradation was evidenced by western blot using an anti-Vt antibody, after fractionation of proteins by 7.5% SDS-PAGE. **(B),** Vt and ovarian homogenates were incubated for 12 h at 37°C in a reaction medium at pH 4.0 either in the absence (lane 3) or in the presence of pesptatin A (inhibitor of aspartic peptidases, lane 4), Na_3_VO_4_ (inhibitor of tyrosine phosphatases, lane 5) or Na^+^/K^+^ tartrate (a broad spectrum phosphatase inhibitor, lane 6). Purified Vt and ovarian homogenate (lanes 1–2, respectively) were incubated in the same conditions and showed as controls. The arrows in the panels indicate the main subunits of purified Vt (Mr ~170 kDa and 174 kDa) visualized as a single immunoreactive band.

The profile of proteolytic fragments of the main vitellin subunits, visualized by western blot, demonstrated that vitellin degradation was pH-dependent, and that extensive proteolysis occurred at pHs ranging from 3.0 to 4.0 (Fig 8A). It was also shown that negligible amounts of vitellin were present in ovarian homogenates (Fig 8A, lane 2).

When the *in vitro* assays were performed at pH 4.0, which is nearly the optimum pH for the activity of DmCatD and acid phosphatase, vitellin was widely degraded and few proteolytic fragments were observed as faint bands in the western blot (Fig 8B, lane 3). However, vitellin proteolysis was strongly inhibited in the presence of pepstatin A (an inhibitor of aspartic peptidases), Na_3_VO_4_ (an inhibitor of tyrosine phosphatase) and Na^+^/K^+^ tartrate (a broad spectrum phosphatase inhibitor) (Fig 8B, lanes 4–6, respectively), showing similar patterns to that of purified vitellin subjected to the conditions of incubation (12 h, 37°C) (Fig 8B, lane 1). NaF, an inhibitor of serine/threonine phosphatase had no effect in preventing vitellin proteolysis. Taken together, these results showed that, at least under our *in vitro* conditions, DmCatD and an acid phosphatase, most likely a tyrosine phosphatase, are required to promote vitellin proteolysis.

## Discussion

In *D*. *maxima*, deprivation of blood meals during post-vitellogenesis led to degeneration of terminal follicles towards an atretic stage and oocyte resorption [27, 28]. In this species, follicular atresia encompasses proteolysis of the main yolk protein, vitellin, but factors involved on its regulation still remain unclear [27]. This finding prompted us to further explore the role of two acid hydrolases, DmCatD and acid phosphatase, in the process of follicular atresia, focusing on their involvement in yolk protein degradation. At transcriptional and translational levels, fat body and ovaries expressed DmCatD at all stages of the reproductive cycle, although ovarian transcripts were upregulated in vitellogenesis. It was also demonstrated that at atretic stages, ovaries displayed the highest levels of DmCatD and acid phosphatase activities. In addition, it was shown that acid phosphatase activity in atretic ovaries corresponded mainly to a tyrosine phosphatase. DmCatD and tyrosine phosphatase were associated with yolk bodies in vitellogenic follicles, displaying a different cellular distribution in atresia. *In vitro* proteolysis assays demonstrated that DmCatD and tyrosine phosphatase were necessary to induce vitellin degradation.

Studies in *Aedes aegypti* demonstrated that cathepsin B and a serine carboxypeptidase are synthesized in the fat body and stored in developing oocytes along with vitellin [43, 44]. Although it has been established that several peptidases are activated for the first time after oviposition to degrade yolk proteins and support embryogenesis [4], results from *C*. *p*. *pallens* suggest that these peptidases are activated before oviposition to promote follicular atresia [19]. In the fat body of *D*. *maxima*, mRNA DmCatD levels were upregulated at pre-vitellogenesis and vitellogenesis. However, in this tissue, relatively similar levels of pro-DmCatD and DmCatD were observed by western blot at pre-vitellogenesis as well as in early and late follicular atresia. In addition, it is worth mentioning that at pre-vitellogenesis, high activity levels of the enzyme were detected in fat bodies. Therefore, it seems possible that at this stage, DmCatD transcripts from the fat body are translated to pro-DmCatD and processed to its active form. In the context of the physiological functions of the fat body throughout the reproductive cycle of *D*. *maxima*, DmCatD may be of relevance at pre-vitellogenesis by processing stored proteins to supply the fat body cells with amino acids until the female has access to a blood meal.

During vitellogenesis, the biosynthetic capacity of the main fat body cells or trophocytes is considerably developed in order to produce large amounts of yolk protein precursors, which in turn will be released to the hemolymph and stored in the oocytes to ensure rapid maturation of eggs [45]. As expected for a yolk protein precursor, DmCatD mRNA levels and the expression of pro-DmCatD were significantly high in the fat bodies sampled at a representative day of the vitellogenic stage. In addition, pro-DmCatD was detected in the hemolymph of females at all reproductive stages, likely reflecting the dynamics between its secretion from the fat bodies and the storage in the oocytes. In the oocytes, pro-DmCatD would be activated early in response to follicular atresia [27] or latter on, during embryogenesis, like other insect peptidases [4, 7, 9, 43]. Interestingly, all the hemolymph samples displayed activity upon a specific substrate of cathepsin D, although in any case an immunoreactive band compatible with the mature peptidase was detected by western blot. Similar findings were reported in the hemolymph of *Bombyx mori* [46]. It is well established that proteolytic activity of cathepsin D is tightly associated with a strong acidic milieu of lysosomes [12]. Moreover, autocatalytic activity of cathepsin D is mostly involved in pro-cathepsin/cathepsin processing [47]. Therefore, it seems very likely that DmCatD activity in the hemolymph of *D*. *maxima* resulted from processing of pro-DmCatD in the reaction medium. The fact that the enzymatic activity increased in vitellogenesis is in agreement with maximal expression of pro-DmCatD in the fat body during this reproductive stage, which in turn would be released to the hemolymph and stored in the oocytes.

During post-vitellogenesis, the lysosomal system in the fat body of females of *A*. *aegypti* plays an important role in degrading the biosynthetic machinery, contributing thus to the remodeling of trophocytes [45]. Moreover, at this stage, vitellogenin synthesis in the fat body and its concentration in the hemolymph sharply declined, and a lysosomal aspartic peptidase was specifically activated [48, 49]. Similarly, in *D*. *maxima*, we have previously shown that protein vitellogenin expression in the fat body decreased at the end of vitellogenic stage [37]. It was also shown that the levels of vitellogenin in the hemolymph significantly decreased during follicular atresia at the post-vitellogenic period [27]. In this work, DmCatD transcripts were detected in the fat bodies at early and late atresia, at the same time that both, protein expression of DmCatD and its activity increased. Considering these observations, it seems plausible that during follicular atresia of *D*. *maxima*, DmCatD in the fat body may be part of the mechanism that operates in the remodeling of the organ in response to switching from vitellogenesis to the post-vitellogenic stage. In agreement with this suggested physiological role, in follicular atresia DmCatD was detected in the cytoplasm of fat body cells (S5B Fig).

In addition to fat body, peptidases like a cystein peptidase of *B*. *mori* can also be synthesized during vitellogenesis by the ovarian follicular cells [6, 46]. In *D*. *maxima*, high DmCatD mRNA levels detected in vitellogenic ovaries suggested that ovarian tissue, most likely follicular cells, contributed to the stored DmCatD in the oocytes. On the other hand, we also found that in ovarian tissue both, pro-DmCatD and DmCatD were detected at all reproductive stages, but the highest DmCatD activity was found at early and late atresia. In insects, follicular atresia and oocyte resorption are integral processes of the operating system which controls the ovarian function. In most instances, oosorption is a reproductive strategy directed to recouping nutrients to increase life span and future reproductive potential [16]. At present, several lines of evidence strongly suggest that during follicular atresia, yolk proteins, mainly vitellin, are subjected to early degradation. Thus, in atretic ovarian follicles of *C*. *p*. *pallens*, an early activation of cathepsin-like peptidases would likely enhance oosorption and degradation of yolk proteins [19]. In the stink bug *Plautia crossota stali*, once oosorption begins, stored vitellin is degraded and released to the hemolymph as small peptides and amino acids [18]. It was reported that, in *R*. *prolixus*, when follicular atresia was triggered by an immunological challenge, the activation of aspartyl and cysteinyl peptidases became a relevant process in early degradation of yolk proteins [24]. It was reported that, in *R*. *prolixus*, when follicular atresia was triggered by an immunological challenge, the activation of aspartyl and cysteinyl peptidases became a relevant process in early degradation of yolk proteins [24]. In *D*. *maxima*, follicular atresia triggered by blood deprivation was characterized by vitellin proteolysis and activation of the ovarian aspartic peptidase DmCatD but unlike *R*. *prolixus*, no activity of cysteine peptidases was detected at early and late atresia [27]. Considering that in *R*. *prolixus* and *D*. *maxima* the causes that elicited follicular degeneration were quite dissimilar, differences in peptidase activation profiles observed in these two related species may likely reveal intrinsic characteristics of each of the insect models. In *D*. *maxima*, gene expression and activity profile of DmCatD in ovarian tissue suggest that this aspartic peptidase plays a role during follicular atresia, probably promoting early yolk protein degradation. Since in *T*. *infestans* and most triatomines basal oocytes undergo resorption during pre-vitellogenesis if females do not ingest a blood meal [21], DmCatD in ovarian tissue of *D*. *maxima* could be also involved in oocyte resorption during pre-vitellogenesis.

In several insect species, yolk enzymes belonging to the acid phosphatase family have been characterized [8, 42, 50–52]. These hydrolases are activated during embryogenesis and seem to be implicated in vitellin dephosphorylation as part of the yolk protein degradation program [4, 53]. Furthermore, there is evidence linking acid phosphatase activation with cellular degeneration and autophagy [28, 54, 55]. In *D*. *maxima*, we showed that acid phosphatase activity in ovarian tissue was significantly elevated at early and late atretic stages. Moreover, by the use of several inhibitors, it was demonstrated that such an activity corresponded mainly to a tyrosine phosphatase. Tyrosine phosphatase activity, as determined by employing specific phosphopeptides as substrates, was significantly high at early atresia and was specifically inhibited by Na_3_VO_4_ as well as by and Na^+^/K^+^ tartrate, a broad spectrum phosphatase inhibitor. However, activity upon a specific substrate of serine/threonine phosphatase was also detected in atretic ovaries. Protein tyrosine phosphatases are involved in several biological processes [56] and its expression or activity during oogenesis and early embryogenesis has been reported in some insect species [52, 57, 58] as well as in the nematode *Ascaris suum* [59]. In addition, insect vitellogenins/vitellins are highly phosphorylated [2] and its dephosphorylation by yolk acid phosphatases appears to be relevant in regulating its breakdown during embryogenesis [9, 10, 60]. Predictions of serine, threonine and tyrosine phosphorylation sites employing NetPhos 2.0 Server (http://www.cbs.dtu.dk/services/NetPhos/) were performed using a partial sequence of vitellin from *D*. *maxima* obtained by tandem mass spectrometry. High confidence putative phosphorylation sites for the three amino acids searched were found (S6 Fig). In this context, we suggest that in *D*. *maxima* both, tyrosine and serine/threonine phosphatases are activated early during follicular atresia to mediate yolk protein degradation as occurred in the embryogenesis of other insect species. Alternatively, activation of acid phosphatase during follicular atresia could be also associated with cellular lysis and removal of degenerating ovarian follicles by autophagy [28].

During embryogenesis, acidification of yolk granules triggers activation of proenzymes and yolk proteolysis [61, 62]. In arthropods, interplay between acid phosphatase and several peptidases on vitellin degradation during embryogenesis has been proposed. Thus, in the hard tick *Boophilus microplus*, a tyrosine phosphatase activity upon vitellin increased its susceptibility to degradation by aspartic peptidases [60]. In the cockroach *Periplaneta americana*, it was shown that an acid phosphatase cooperates with a cystein peptidase to degrade vitellin during early embryogenesis [9]. Moreover, in *R*. *prolixus*, cathepsin D activity depended on the activation of an acid phosphatase also present in yolk bodies [10]. Although the association among acid hydrolases is unclear, it has been recently suggested that inorganic polyphosphate stored in yolk vesicles is a substrate for acid phosphatase as well as an inhibitor of yolk peptidases [11, 58]. On the contrary, acid phosphatase and cathepsin B activities followed similar patterns during embryogenesis of *Musca domestica*, but inhibition of acid phosphatase did not affect cathepsin action towards yolk proteins [51]. In this work, we showed that acid phosphatase and DmCatD were activated early during follicular atresia of *D*. *maxima*. *In vitro* proteolysis assays using homogenates of atretic ovaries as enzyme source and specific inhibitors allowed us to demonstrate that vitellin proteolysis was pH-dependent and most importantly, that acid phosphatase and DmCatD were necessary to promote vitellin degradation.

The spatial localization of peptidases and substrates is an important factor in controlling yolk protein degradation [4]. It was reported that in some insect species, peptidases found in yolk bodies were localized in the non-crystaline matrix of such structures, separated from yolk proteins [44]. A cathepsin D-like peptidase appeared to be bound to yolk body membranes in *R*. *prolixus* [8]. In *B*. *mori* oocytes, a cystein peptidase was localized in small sized yolk bodies, separated of the yolk proteins [63]. On the other hand, there are reports suggesting that acid phosphatase activity presented some compartmentalization in ovarian follicles, being found in small vesicles of the oocytes but not inside yolk bodies [52, 58]. In vitellogenic females of *D*. *maxima*, tyrosine phosphatase and DmCatD were distributed in the periphery of yolk bodies, partially co-localizing with vitellin, suggesting a compartmentalization to limit vitellin proteolysis during oogenesis. Interestingly, at early atresia, activities of acid phosphatase and DmCatD peaked and both enzymes changed their distribution pattern, along with the loss of organized yolk bodies. FRET assays demonstrated that in mature yolk bodies, vitellin was at a 1–10 nm distance from tyrosine phosphatase and DmCatD, facilitating their interaction during the stage of follicular atresia. On the other hand, it is worthy of note that in atretic follicles, fluorescent signal for DmCatD was also observed in the basal domains of follicular cells, probably because of its involvement in cell death by apoptosis and autophagy [28]. Besides, tyrosine phosphatase signal was detected in the perioocytic space, a localization that might indicate its transit to the yolk bodies of the oocyte. Alternatively, the enzyme can be acting on the plasma membrane of the oocyte, modifying its phospholipids [4].

Taken together, the results demonstrated for the first time that in *D*. *maxima*, DmCatD is synthesized by the fat body and ovarian tissue, although the regulatory mechanism involved in such a synthesis has not been established yet. It was also shown that *in vitro*, DmCatD and acid phosphatases, mainly a tyrosine phosphatase, are involved in vitellin proteolysis during follicular atresia. Early activation of acid phosphatases and DmCatD in atretic ovaries of triatomines may be of physiological relevance regulating yolk protein degradation to sustain younger oocytes until the improvement of nutritional conditions. In fact, under standardized conditions, females of *D*. *maxima* can take a second blood meal around days 5–7 after the end of oviposition period to resume vitellogenesis successfully. Future research directed to elucidate the mechanisms by which both acid hydrolases contribute to vitellin degradation would be necessary to achieve a better characterization of the process of follicular atresia, a key event that impacts on the life cycle of triatomine females and consequently, on the epidemiology of Chagas’ disease.

## Supporting Information

S1 Fig The dissociation curves (melting curves) of DmCatD (A) and 18S ribosomal RNA (B) qPCR products analyzed in fat body and ovarian tissues throughout the reproductive cycle of *D*. *maxima*. As indicated by a single dissociation curve peak, each pair of primers on qPCR produced a single specific product in both tissues at all stages of the reproductive cycle.
(TIF)

S2 Fig Ponceau S staining profiles of western blots. Fat body (A) and ovarian homogenates (B) were subjected to SDS-Tris-Tricine gel electrophoresis, transferred onto nitrocellulose membranes and stained with Ponceau S in order to perform densitometric analyses of immunoreactive bands detected on western blots. The blot shown in the Figure is a representative experiment of three independent assays.
(TIF)

S3 Fig Size and specificity of DmCatD PCR product. (A) Amplification specificity of DmCatD mRNA by 2% agarose gel electrophoresis. A single PCR product of the expected size was amplified. Lanes represent: 1, DmCatD; 2, molecular weight marker (50 bp ladder). (B-C) Sequencing of the PCR product. The specificity was confirmed by automatic sequencing (MACROGEN, Seoul, Korea) and subsequent analysis using the program BLAST (Basic Local Alignment Search Tool). For the analyses, the tool Blastx was employed to find potential matches with DmCatD. The analyzed data suggested a match with cathepsin D of *Triatoma infestans*, a closely related triatomine.
(TIF)

S4 Fig DmCatD expression analyzed by RT-PCR in fat bodies (A) and ovarian tissues (B) of *D*. *maxima* at different stages of the reproductive cycle. Dissected fat bodies and ovaries of three females were pooled and RNA extraction was performed employing the MasterPure RNA Purification Kit according to the manufacturer´s protocol. RNA integrity was evaluated by electrophoresis in a 1% agarose gel electrophoresis. The specificity was confirmed by automatic sequencing (MACROGEN, Seoul, Korea). PCR products were separated on a 2% agarose gel electrophoresis and visualized with ethidium bromide. 18S ribosomal RNA was used as internal control.
(TIF)

S5 Fig DmCatD localization in fat body by indirect immunofluorescence assay. Fat body cryosections obtained from females at vitellogenesis (**A**) and early atresia (**B**) were incubated with the commercial anti-cathepsin D antibody (dilution 1:100) followed by Alexa Fluor 568-conjugated goat anti-rabbit IgG antibody (dilution 1:1000) as stated in Materials and Methods. Thereafter, slides were incubated with 300nM DAPI in PBS in a humid chamber (37°C, 40 min in the dark). Slides were mounted in Fluorsave and viewed with an Olympus Fluoview 300 laser scanning confocal microscope. The fluorescent signal for DmCatD in vitellogenic and atretic fat bodies (A-B, respectively) was distributed thoroughly in the cytoplasm of the trophocytes (arrows). Similar results were obtained in three separate experiments. Bars: 10 μm.
(TIF)

S6 Fig Predictions of serine, threonine and tyrosine phosphorylation sites of vitellin tryptic peptides. Phosphorylation sites were predicted employing NetPhos 2.0 Server (http://www.cbs.dtu.dk/services/NetPhos/) using tryptic peptides of *D*. *maxima* vitellin sequenced by electrospray ionization quadrupole time-of-flight mass spectrometry. Only high confidence putative phosphorylation sites for the three amino acids searched are shown (arrows).
(TIF)
