# Supplementary figures and images for: The Role of DmCatD, a Cathepsin D-Like Peptidase, and Acid Phosphatase in the Process of Follicular Atresia in Dipetalogaster maxima (Hemiptera: Reduviidae), a Vector of Chagas' Disease
Source: PLoS One. 2015 Jun 19;10(6):e0130144. doi: 10.1371/journal.pone.0130144 (PMC4474837; doi:10.1371/journal.pone.0130144)

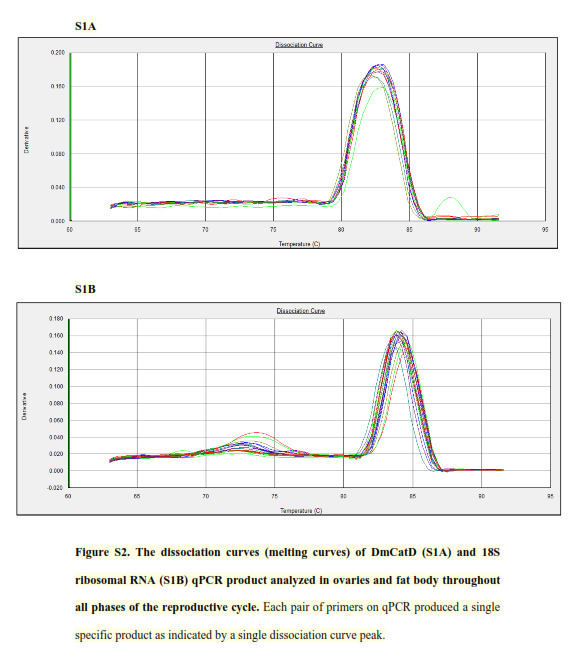

Supplement: S1 Fig — As indicated by a single dissociation curve peak, each pair of primers on qPCR produced a single specific product in both tissues at all stages of the reproductive cycle. (TIF) [file pone.0130144.s001.tif]

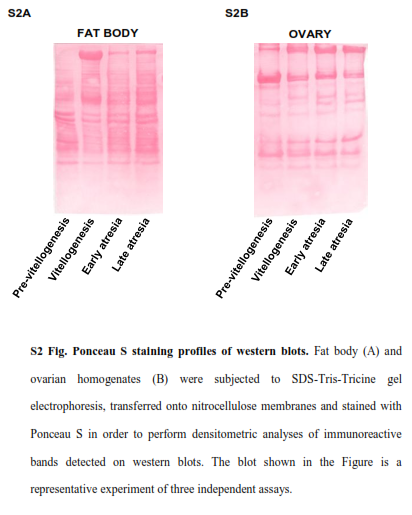

Supplement: S2 Fig — Fat body (A) and ovarian homogenates (B) were subjected to SDS-Tris-Tricine gel electrophoresis, transferred onto nitrocellulose membranes and stained with Ponceau S in order to perform densitometric analyses of immunoreactive bands detected on western blots. The blot shown in the Figure is a representative experiment of three independent assays. (TIF) [file pone.0130144.s002.tif]

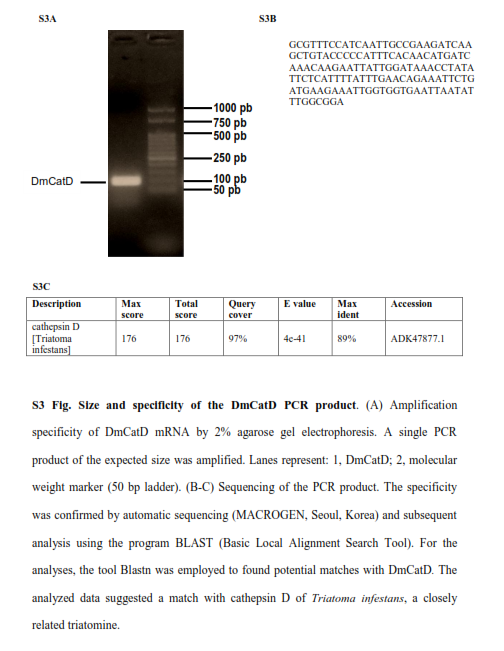

Supplement: S3 Fig — (A) Amplification specificity of DmCatD mRNA by 2% agarose gel electrophoresis. A single PCR product of the expected size was amplified. Lanes represent: 1, DmCatD; 2, molecular weight marker (50 bp ladder). (B-C) Sequencing of the PCR product. The specificity was confirmed by automatic sequencing (MACROGEN, Seoul, Korea) and subsequent analysis using the program BLAST (Basic Local Alignment Search Tool). For the analyses, the tool Blastx was employed to find potential matches with DmCatD. The analyzed data suggested a match with cathepsin D of Triatoma infestans, a closely related triatomine. (TIF) [file pone.0130144.s003.tif]

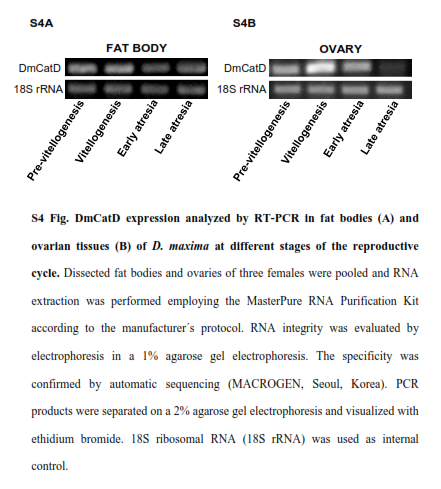

Supplement: S4 Fig — Dissected fat bodies and ovaries of three females were pooled and RNA extraction was performed employing the MasterPure RNA Purification Kit according to the manufacturer´s protocol. RNA integrity was evaluated by electrophoresis in a 1% agarose gel electrophoresis. The specificity was confirmed by automatic sequencing (MACROGEN, Seoul, Korea). PCR products were separated on a 2% agarose gel electrophoresis and visualized with ethidium bromide. 18S ribosomal RNA was used as internal control. (TIF) [file pone.0130144.s004.tif]

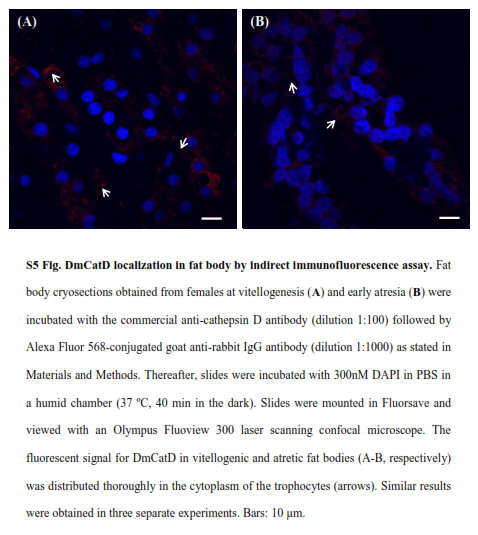

Supplement: S5 Fig — Fat body cryosections obtained from females at vitellogenesis (A) and early atresia (B) were incubated with the commercial anti-cathepsin D antibody (dilution 1:100) followed by Alexa Fluor 568-conjugated goat anti-rabbit IgG antibody (dilution 1:1000) as stated in Materials and Methods. Thereafter, slides were incubated with 300nM DAPI in PBS in a humid chamber (37°C, 40 min in the dark). Slides were mounted in Fluorsave and viewed with an Olympus Fluoview 300 laser scanning confocal microscope. The fluorescent signal for DmCatD in vitellogenic and atretic fat bodies (A-B, respectively) was distributed thoroughly in the cytoplasm of the trophocytes (arrows). Similar results were obtained in three separate experiments. Bars: 10 μm. (TIF) [file pone.0130144.s005.tif]

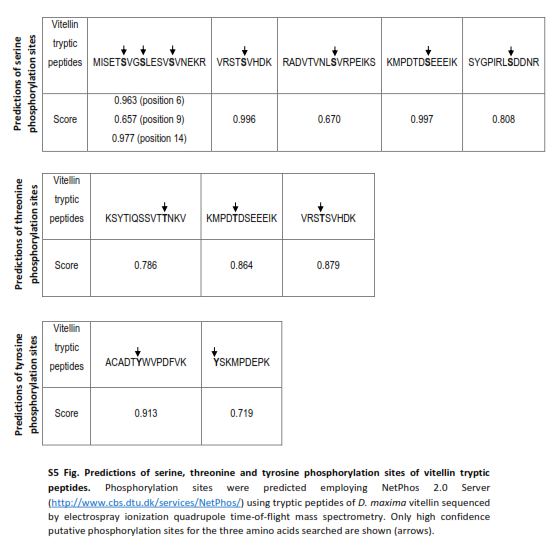

Supplement: S6 Fig — Phosphorylation sites were predicted employing NetPhos 2.0 Server (http://www.cbs.dtu.dk/services/NetPhos/) using tryptic peptides of D. maxima vitellin sequenced by electrospray ionization quadrupole time-of-flight mass spectrometry. Only high confidence putative phosphorylation sites for the three amino acids searched are shown (arrows). (TIF) [file pone.0130144.s006.tif]
